# Effects of stevia on glycemic and lipid profile of type 2 diabetic patients: A randomized controlled trial

**Published:** 2020

**Authors:** Marjan Ajami, Maryam Seyfi, Fatemeh Abdollah Pouri Hosseini, Parisa Naseri, Aynaz Velayati, Fahimeh Mahmoudnia, Malihe Zahedirad, Majid Hajifaraji

**Affiliations:** 1 *Department of Food and Nutrition Policy and Planning Research, National Nutrition and Food Technology Research Institute, Shahid Beheshti University of Medical Sciences, Tehran, Iran*; 2 *Nutritional Sciences, National Nutrition and Food Technology Research Institute, School of Nutrition Sciences and Food Technology, Shahid Beheshti University of Medical Sciences, Tehran, Iran*; 3 *Department of Biostatistics, School of Allied Medical Sciences, Shahid Beheshti University of Medical Sciences. Tehran. Iran*; 4 *Department of Biology, Faculty of Science, Farhangian University, Tehran, Iran*; 5 *Department of Nutrition, School of Nutrition and Food Technology, Shahid Beheshti University of Medical Sciences, Tehran, Iran*

**Keywords:** Stevia, Sucralose, Type 2 diabetes, Glycemic response, Lipid profile

## Abstract

**Objective:**

Stevia (*Stevia rebaudiana Bertoni*) is a natural and healthy alternative sweetener to sugar and artificial sweeteners, which has become important for human diets and food manufactures. In this study, the effects of stevia or sucralose as tea sweeteners on glycemic and lipid profile of type 2 diabetic patients were investigated.

**Materials and Methods:**

A double-blind clinical trial was carried out in 34 type 2 diabetic patients. These patients were assigned into two groups of stevia (n=15) (received 1 cup of 2% stevia extract-sweet tea in three meals) and non-stevia (n=19) (received one tablet of sucralose sweetener) daily for eight weeks. Glycemic response and lipid profile of the participants were assessed. Furthermore, height, weight and body mass index (BMI) of the participants were measured as well as their dietary intakes at the baseline and at the end of the study.

**Results:**

Findings showed no significant differences in fasting blood sugar (FBS) levels between the base line and after two hours, in participants. Also, no significant differences in insulin, glycosylated hemoglobin (HbA1C) and lipid levels were found between the two groups.

**Conclusion:**

Results of the current study showed that the highlighted doses of stevia in sweetened tea could be an alternative to sucralose in diabetic patients with no effects on blood glucose, HbA1C, insulin and lipid levels.

## Introduction

Low-calorie foods with low sugar contents are being fast developed by the food industries to prevent obesity and metabolic syndromes in consumers. Various artificial sweeteners that have been suggested as substitutes for sugar in foods and beverages are associated with protective metabolic effects such as lower sugar and calorie intakes (Anton, 2010 ▶). These effects play a significant role in obese individuals and diabetic patients (Lozano, 2010 ▶; Azimi-Nezhad, 2008 ▶). Sucralose, aspartame, saccharin and acesulfame potassium are some commercially available artificial sweeteners which are widely used as calorie-free alternatives to sugars. Although these sweeteners are approved as safe, they are classified as non-nutritive sweeteners and subjected to controversy due to chemical additives. Recent studies suggested that artificial sweeteners contribute to weight gain and hence increase the risk of metabolic syndromes, cardiovascular diseases (CVD) and type 2 diabetes (Tandel, 2011 ▶; Swithers, 2013 ▶). However, artificial and non-nutritive sweeteners exert benefits in management of diabetes, previous studies did not provide substantial evidence if the use of these sweeteners maintains normal blood glucose levels or possesses any effects on weight management. Therefore, herbal sweeteners such as stevia (*Stevia rebaudiana*) are receiving much attention as natural alternatives to artificial sweeteners and sugars, especially in management of insulin sensitivity and type 2 diabetes (Reid, 2016 ▶; Romo-Romo, 2016 ▶). Stevia has antioxidant and anti-inflammatory properties (Ruiz Ruiz, 2014 ▶), which can be used for treatment of oxidative stress-linked tissue pathologies (Xu, 2008 ▶). Stevia, was used to attenuate tissue damage after ischemia and metabolic stresses in various body organs (Xu, 2007 ▶, 2008; Chavushyan, 2017 ▶; Potocnjak, 2017 ▶). Steviol is the major constitute of glycosides in stevia and gives the herb’s sweet taste. Stevia contains high quantities of diterpene glycosides, which cannot be broken down or absorbed by the digestive tract. Therefore, intake of stevia sweetener does not affect the blood glucose level. Unlike the low-calorie synthetic sweeteners, stevia is quite safe, non-toxic and non-mutagenic; also, it is 200-times sweeter than the regular sugar (sucrose) and calorie-free. Daily intake of 2 mg/kg/bw of stevia was reported to be safe, especially in diabetic patients (Prakash, 2017 ▶; Abo Elnaga, 2016 ▶; Sharma, 2016 ▶). Stevia was approved by the Codex commission, including the World Health Organization (WHO) and Food Agriculture Organization (FAO). Furthermore, standard steviol glycoside solutions and doses have been set by the Joint FAO/WHO Expert Committee on Food Additives (JECFA) (WHO, 2006 ▶). By the end of 2004, cultivation of stevia began in Iran in forms of tissue cultures. By 2006, various species of stevia seedlings (laboratory samples) were planted and their growth compatibility was assessed. This resulted in commercialization and development of the plant in the north of Iran. In Iran, commercial production of stevia began in 2008. The industrial products of this natural sweetener are likely to replace a large portion of regular sugars in the near future (Karimi, 2014 ▶). Nowadays, stevia is commercially cultivated in Paraguay, Brazil, Central America, China, Thailand and USA. Moreover, stevia is widely used as a sweetener in Japan and South Korea. Animal studies and clinical trials in Brazil, England and Japan suggested that stevia can regulate blood sugar level (Yadav, 2011 ▶; Goyal, 2010 ▶). In Paraguay, stevia-sweetened tea is used for regulation of blood glucose. To some extent, stevia can decrease high levels of blood sugar, however, it shows no lowering effects on normal levels of blood sugar (Misra, 2011 ▶). Since tea is one of the most popular hot drinks in the world, the aim of the current study was to investigate if glycemic and lipid profile of diabetics patients were changed after drinking stevia- or sucralose-sweetened tea. 

## Materials

**Study design and participants**

A double-blind randomized clinical trial was carried out to compare the effects of stevia- and sucralose-sweetened teas in 39 eligible type-2 diabetic patients, who were randomly assigned into two groups (19 in stevia and 20 in control groups). Five patients chose to withdraw from the study and 34 patients completed the study. The inclusion criteria included fasting blood sugar (FBS) level <180 mg/dl, postprandial glucose (PPG) level <250 mg/dl, glycosylated hemoglobin (HbA1C) value <10% (Gregersen, 2004 ▶), BMI between 18.5 and 29.9 kg/m^2^ and no pregnancy, lactation or insulin dependency. Patients with conditions such as autoimmune disorders, chronic inflammatory diseases, ischemic heart problems, renal disorders and thyroid disorders and those allergic to stevia were not included in the study; participants were questioned and assessed in person and via phone calls concerning the exclusion criteria such as continuous and daily intake of tea, occurrence of any clinical complications associated to stevia intake, alcohol consumption, drug use, changes in routine diabetes treatments, pregnancy and endocrine, cardiac, neurological and renal diseases during the study. The participants’ weights and heights were recorded using portable digital scale (Seca, Germany) with 10-g accuracy and non-elastic tape measure with 0.1-cm accuracy, respectively. Informed consent forms were signed by the participants before initiating the study. The participants were randomly assigned into two major groups of stevia and sucralose. First, 5 ml of blood was collected from forearm veins of the participants to assess complete blood count (CBC), and glycemic and lipid profiles before the intervention. Following a 12-hour fasting period, the participants were provided with a simple breakfast, including 60 g of branny bread, low-salt cheese and 200 ml of black tea sweetened with either 2% of stevia extract or sucralose tablet. The tea bags were provided for a daily use of the subjects within eight weeks. Stevia was collected from domestic cultures and stevia extract was produced and added to the teabags by Parmida Mehr Pasargad, Iran. The product received national patent (Ref. A-89/013612) and was approved by Iran Food and Drug Administration (IFDA) for the safety of physical, chemical, microbial and toxicological characteristics (Ref. 675/134968). Then, 2 ml of blood was collected from the forearm veins of the participants at the baseline and weeks 4 and 8 of the study, to determine FBS and PPG (2 hours after intake) levels as well as fasting HbA1c, and glycemic and lipid profiles. The participants were asked to assess their blood FBS and 2-hour PPG three times a week using portable glucometers. Dietary intakes of the participants were recorded using 24-hour recall questionnaires for three days (twice during the week and once at the weekend) at the beginning and the end of the study. The participants were also asked to keep on their restricted diets having at least mild daily physical activity and inform the investigators about any changes in their medicine during the study. Nutritionist IV software v.3.5.2 was used to carry out the diet analysis. To calculate the sample size, a variance in glucose levels of 1.5 mg/dl and a δ of 0.6 were considered. Study was approved by the Research Ethics Committee of National Nutrition and Food Technology Research Institute, Iran, (Reference No. IR.SBMU.nnftri.Rec.1394.23) and registered in the Iranian Registry of Clinical Trials (Reference No. 28816). 

**Laboratory tests**

Blood glucose levels were assessed through enzymatic methods using D-glucose (GOD) colorimetric kits. Blood total cholesterol (TC) was assessed using Trinder CHOD/POD End Point enzymatic method and blood triglycerides (TG) using GPO-PAP (Glycerol-3-phosphate oxidase producing hydrogen peroxide) method. HbA1C was assessed through boronic acid tendency method using NORUDIA^™^ N HbA1c kit (Sekisui Medical, Japan). Fasting insulin levels were assessed by a chemiluminescent immunoassay using AccuBind^™^ ELISA Microwells kit (Monobind, USA). Insulin resistance was assessed using HOMA-IR formula. FBS and PPG levels were assessed twice a week (with 2-day intervals) using ACCU-Chek Active™ glucometer (Roche, Switzerland). 

**Statistical analysis**

Descriptive statistics for all numerical variables are presented as mean±SD (standard deviation). All results were assessed for normality using Kolmogorov-Smirnov statistical test. Paired and independent t-tests were used for the analysis of differences between the mean HbA1C levels of the two groups at the baseline and on day 60. Differences in glycemic profiles of the two groups were compared at the baseline and following various time intervals (days 30, 60, 90 and 120) using marginal model of generalized estimation equation (GEE). Moreover, Bonferroni’s *post hoc* test was used to analyze and compare the glycemic responses (in a pairwise multiple comparison). All analyses were carried out using SPSS software v.23 (IBM Analytics, USA) and p values less than 0.05 were considered statistically significant. 

## Results

The current study started in 2016 and completed in 2017. Of 39 diabetic patients, five (three from the experimental and two from the control groups) were excluded due to uncompleted sessions. No significant differences were found in age of the participants between the two groups (p=0.217). The BMI values of the two groups were compared to each other based on negating the sphericity assumption using Greenhouse-Geisser adjustment. Intra-group comparisons were carried out based on the sphericity assumption with a p value of 0.02 in the stevia group VS the sucralose group (p=0.61), for a significant decrease in BMI. No significant differences were found in mean systolic and diastolic blood pressures between the two groups. Differences in waist circumferences of the two groups were statistically significant at the beginning of the study (p=0.031); 103.0±12.04 and 95.4±8.32 cm in stevia and sucralose groups, respectively. However, waist circumference did not vary significantly between the two groups based on the sphericity assumption (p=0.58). 

**Table 1:** Demographic characteristics of the participants in stevia and sucralose groups

**Group**		**Stevia**	**Sucralose**
**Sex (%)**	**Male**	33.3	38.1
	**Female**	66.7	61.9
**Age (year)**		55.3±7.4	52.1±7.6
**WC (cm)**		103.0±12.04^*^	95.4±8.32
**BMI (kg/m** ^2^ **)**		30.87±6.32	27.51±3.04
**SBP**		13±1.9	12.9±1.7
**DBP**		7.4±0.9	7.4±1.0

WC, Waist circumferences; BMI, Body mass index; SBP, Systolic blood pressure; DBP, Diastolic blood pressure; ^*^p<0.05, significant

Average energy and macronutrient intakes of the participants drinking stevia- or sucralose-sweetened tea, before the start of the intervention is shown in Table 2. This was carried out to assess the data consistencies or changes within the groups. The mean changes within the two groups are demonstrated in Table 2. The GEE marginal test was used to assess treatment effects on blood parameters (i.e. glycemic and lipid profiles) between the two groups at various time points (baseline, and days 30 and 60). No significant differences were reported by the adjustment of confounding variables such as time and sex. Independent t-test was used to compare the baseline and day 60 values between the two groups. 

HbA1C test was used for comparing changes in average hemoglobin levels between the two groups but no significant differences were observed (Table 3). 

**Table 2:** Comparison between the effects of stevia- and sucralose-sweetened teas on energy and macronutrient intakes of the participants

**Variable**	**Sucralose**			***P*** ** value** ^1^	**Stevia**			***P*** ** value** ^2^	***P*** ** value** ^3^	***P*** ** value** ^4^
	**Baseline**	**End**	**Average difference**		**Baseline**	**End**	**Average difference**			
**Energy (kcal) **	1520.57±606.97	1725.00±595.06	204.43±154.95	0.20	1603.93±848.52	1379.71±477.56	-224.21±284.12	0.44	0.75	0.20
**Protein (g)**	50.43±13.87	69.57±31.86	19.14±7.71	0.02	64.14±48.02	54.43±14.79	-9.71±13.87	0.49	0.33	0.06
**Carbohydrate (g)**	227.41±111.24	242.90±113.20	15.49±24.52	0.54	227.44±136.09	196.91±58.67	-30.53±39.66	0.45	1.00	0.30
**Simple sugar (g)**	15.79±10.16	13.54±6.97	-2.25±1.55	0.16	25.11±24.20	17.62±11.93	-7.94±6.39	0.26	0.21	0.44
**Fat (g) **	48.44±31.17	55.41±21.20	6.97±7.61	0.37	51.74±29.72	44.26±32.04	-7.48±14.15	0.60	0.76	0.33
**Saturated fatty acid (g)**	12.24±4.87	14.06±8.32	3.29±1.32	0.23	14.06±8.32	8.45±3.29	-5.61±2.80	0.06	0.056	0.01
**Unsaturated fatty acid (g)**	15.00±14.79	19.92±11.99	4.92±3.98	0.23	16.19±12.02	12.29±11.56	-3.91±5.40	0.48	0.81	0.18

p Value^1,2^, calculated using paired *t-*test for changes within the groups

p value^3^, calculated using independent *t*-test to compare the mean of the two groups at the beginning of the study (coincidence)

p value^4^, calculated using Independent *t*-test to compare the mean of changes between the two groups after the intervention

**Table 3:** Comparison between the effects of stevia- and sucralose-sweetened teas on glycemic response and lipid profile of the participants

Variable	**Sucralose (n=19)**			**Stevia (n=15)**			**p value**
Day	**Baseline**	**30**	**60**	**Baseline**	**30**	**60**	
**Fasting blood sugar (mg/dl)**	149.35 ±46.1	157.90 ±50.67	161.94 ±42.42	157.46 ±58/14	161.46 ±53.73	160.71 ±50.91	0.45
**Postprandial blood sugar (2-hour) (mg/dl)**	198.2 ±55.75	204.40 ±61.75	218.76 ±50.82	212.8 ±81.46	204.60 ±66.03	212.57 ±64.95	0.32
**Insulin (mU/I)**	7.95 ±3.31	7.82 ±3.41	7.49 ±3.18	10.81 ±6.49	10.01 ±4.98	8.91 ±4.13	0.29
**Total cholesterol (mg/dl)**	150.85 ±25.61	149.35 ±28.63	157.65 ±28.56	162.13 ±44.72	179.20 ±36.22	170.57 ±35.56	0.85
**Triglyceride (mg/dl)**	136.82 ±54.66	112.29 ±47.9	144.17 ±74.32	179.71 ±99.65	170.50 ±110.78	148.21 ±73.43	0.1
**LDL cholesterol (mg/dl)**	78.75 ±18.07	81.90 ±18.11	83.58 ±19.25	87.53 ±23.20	94.93 ±16.61	94.92 ±20.64	0.66
**HDL cholesterol (mg/dl)**	51.65 ±14	40.30 ±12.16	45.00 ±14.79	52.13 ±10.32	50.93 ±11.18	48.57 ±7.79	0.28
**Glycosylated hemoglobin **	6.95 ±1.16		6.93 ±1.13	7.07 ±1.67		6.89 ±1.32	0.53

^δ^ Repeated measures test was used to analyze intra-group variations of the variables

Paired t-test was used for evaluation of intra-group changes and independent t-test to make comparison of mean changes between the two groups

**Figure 1:**
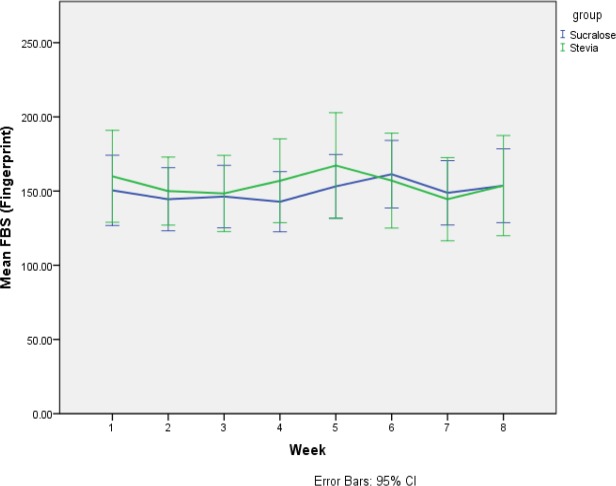
Comparison of FBS mean differences between the stevia and sucralose groups

**Figure 2:**
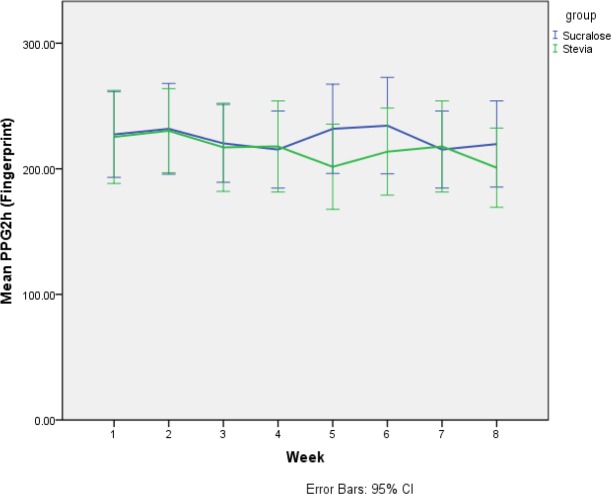
Comparison of 2-hour PPG mean differences between the stevia and sucralose groups

Changes in FBS and 2-hour PPG levels of the participants were assessed using glucometers (carried out by the participants) during eight weeks. Greenhouse-Geisser assumption was used for the comparison of differences between the stevia and sucralose groups using repeated assessments and rejected null hypotheses with no significant differences reported (p values of 0.5 and 0.75, respectively). Using sphericity assumption, intra-group changes were assessed in stevia (p value=0.99) and sucralose (p value=0.06) groups and showed no significant differences. Despite no significant differences between the two groups, PPG levels of participants were more satisfactory in response to stevia than sucralose. Figures 1 and 2 show differences in the FBS and 2-hour PPG levels of the participants between the two groups. Repeated assessments of the participants’ FBS and 2-hour PPG levels showed no significant differences between the stevia and sucralose groups by rejection of sphericity assumption and use of Greenhouse-Geisser adjustment (p values of 0.50 and 0.75, respectively). Moreover, sphericity assumption revealed no significant differences in FBS (p=0.99 instead of p=0.06) and 2-hour PPG (p=0.88 instead of p=0.16) between stevia and sucralose groups. The TC levels increased in both groups at the end of the study. However, differences were not significant between the two groups when adjusted for the baseline values and confounding variables such as time and sex.

## Discussion

The current study was carried out to assess effects of stevia on glycemic and lipid profiles of type 2 diabetic patients. Results showed no statistically significant differences between the highlighted blood parameters in both groups of diabetic patients after two months. Insulin, FBS, HbA1c, PPG, TG, TC, LDL and HDL did not change significantly in both groups.

. Importance of stevia as a healthy and natural alternative to sugar and artificial sweeteners, is rising within the societies. Furthermore, stevia shows a high heat stability, which is a favorable characteristic for the food industries. Interestingly, stevia is nearly 250–300 times sweeter than regular sugar (sucrose) and non-nutritive sweeteners with no calories and complications (Brown, 2010 ▶). Extra calories usually result in weight gains and other metabolic problems such as insulin resistance in consumers. Controversial data exist on the effectiveness of artificial sweeteners for the management of diabetes (Gardner, 2012 ▶). In fact, stevia does not contribute to increased glucose and insulin levels. One of the most important biological effects of stevia is opening calcium channels in pancreatic beta cells mediated by the active constitute of *S.* *rebaudiana*, steviol*.* This stimulates insulin secretion in response to glucose. Therefore, use of stevia for the management of type 2 diabetes is recommended (Momtazi-Borojeni, 2017 ▶). In 2017, Philippaert et al. reported the benefits of stevia for healthy individuals as a preventive mechanism against diabetes (insulin resistance), especially for those who are on high fat diets (Philippaert, 2017 ▶).

In the current study, changes in blood glucose were first assessed following the consumption of stevia-sweetened tea due to the importance of glycemic response in diabetic patients. Lack of significant changes in blood glucose levels in the current study was similar to that observed in a study done by Genus et al. They found that oral intake of 250 mg of stevioside (three times a day) for one year, did not affect blood glucose levels in healthy individuals (Geuns, 2007 ▶). In the present study, consumption of 2% stevia-sweetened tea (one or three times daily for two months) contributed to significant changes in FBS and HbA1c levels in diabetic patients with no statistically significant differences, compared to the control (sucralose group). Because the life span of red blood cells (RBCs) is nearly 120 days, a 50% turn over in a 2-month period can be judged; however, routine assessment is often carried out every three months. Moreover, consumption of stevia did not change PPG levels in diabetic patients. In contrast, Awney et al. (2011) ▶ reported significant decreases in blood glucose levels following administration of 41% stevioside solutions (Awney, 2011 ▶). This inconsistency might occur due to low doses of stevia in tea bags used in the current study. Therefore, low doses of stevia do not likely cause significant changes in glycemic responses as previously expected. For example, stevioside was reported to regulate blood glucose levels in diabetic rat models by increasing insulin secretion through downregulation of phosphoenolpyruvate carboxykinase (PEPCK) gene expression. The PEPCK protein is an enzyme that activates the metabolic pathway of gluconeogenesis and converts oxaloacetate into phosphoenol pyruvate and carbon dioxide. Therefore, inhibition of this enzyme or reduction in its gene expression can decrease glucose production from non-sugar sources (Awney, 2011 ▶; Geeraert, 2010 ▶).

Despite expectations, stevia did not cause significant changes in lipid profile of diabetic patients. Of the analyzed parameters, no significant changes were found in TG, LDL and HDL. These findings were different from those reported by Elnaga et al. (2016) in a study on the effects of stevia on body weight and other biochemical parameters. Compared to sucralose, stevia was reported to decrease body weight and blood TG, LDL and TC levels but increase HDL levels in rats (Abo Elnaga, 2016 ▶). However, no significant changes were found in blood HDL levels in both groups of the current study. These differences could be attributed to dietary intakes of the participants. Participants were instructed not to change their physical activity or dietary patterns during the intervention (8 weeks). Furthermore, energy, carbohydrate, fat and protein intakes were calculated based on 24-hour recalls before and after the intervention. Diet analysis of the participants showed that dietary protein significantly increased in sucralose group while dietary saturated fat was significantly decreased in stevia group at the end of the intervention. Since energy intake was nearly similar in both groups at the beginning and end of the study, it might be suggested that sweeteners did not affect the participants’ appetite. It is noteworthy that the average energy increased from 1520.57±606.97 to 1725±596.085 in sucralose group but decreased from 1603.93±848.52 to 1379.71±477.56 in stevia group with no statistical significances. Furthermore, BMI of the participants significantly decreased in stevia group, which could be explained by decreased energy intakes. In 2010, Geeraert et al. reported that oral intake of stevia for 12 weeks did not produce any effects on body weight but significantly decreased blood glucose and insulin levels, compared to the placebos group. However, a two-fold increase in blood adiponectin levels was associated with increases in insulin signaling and antioxidant defense in vascular walls of the adipose tissue (Geeraert, 2010 ▶). 

Evidently, stevia plays important roles in improvement of glycemic response and lipid profile in contrast to artificial sweeteners (e.g. sucralose) (Talevi, 2017 ▶). Another advantage of stevia is linked to increased insulin sensitivity and hence, this herb can be helpful in management of type 2 diabetes (Anton, 2010 ▶). Moreover, stevia not only induces a low glycemic response, but also provides essential nutrients such as vitamins A, B3 and C and minerals including magnesium, potassium, selenium and zinc. Indeed, the antioxidant and antimicrobial properties of stevia as well as its high heat stability make this natural sweetener favorite for the food industries (Lemus-Mondaca, 2012 ▶). However, further studies are recommended during longer periods using various doses of stevia. Antioxidant and anti-inflammatory properties of stevia and other food supplements such as omega-3 fatty acids, were reported to decrease apoptotic cell death and tissue damage after ischemia, oxidative stress and metabolic stress with relatively few side effects (Ajami, 2013, 2011).

The current study was carried out to investigate potential protective effects of stevia on blood parameters in diabetic patients. Use of stevia (as a natural sweetener) resulted in no significant differences in glycemic response and lipid profile of type 2 diabetic patients, compared to sucralose (as an artificial sweetener) did. No significant differences were seen in blood insulin, glycosylated hemoglobin and lipid levels after the use of stevia or sucralose. In conclusion, it seems that the of stevia as a natural sweetener produce no significant metabolic effects at specific dose described in the manuscript. Further studies are necessary to investigate long-term effects of stevia on human health as well as its effective doses. 
